# Using System Dynamics for Local Authority Public Health practice in England

**DOI:** 10.23889/ijpds.v9i5.2895

**Published:** 2024-09-18

**Authors:** Abraham George

**Affiliations:** 1 Kent County Council

A key function of local authority public health teams in England is to undertake a regular health needs assessment of their population. This involves forecasting, among other things, prevalence and incidence of long term conditions and health care service demand. Over the last few years, Kent County Council Public Health has adopted the use of system dynamics modelling (SDM) to not only do this but generate scenarios to simulate the impact of different interventions across different cohorts defined by age groups and diagnosable conditions. These include “no conditions” as well as understand the combined effects of different interventions, demographic changes and wider health determinants. Risk factors, such as smoking, diet and physical activity, were calculated using local linked data for the incidence of single conditions, progression toward complex needs and levels of morbidity including frailty. These were used to create the dynamics of the model. Incidence, prevalence and mortality for each cohort were projected over 25 years with “do nothing” and “Better Health” scenarios. The size of the “no conditions” cohort increased, and the other cohorts decreased in size. The impact of the interventions on life expectancy at birth and healthy life expectancy was significant. SDM heavily influenced local authority commissioning decisions for health improvement services redesign such as smoking cessation, weight management as well as other secondary prevention measures such as hypertension management and cardiovascular disease screening delivered by General Practice and other health service partners.

